# Similar PROMs and Healthcare Utilization in Patients Undergoing Total Knee Arthroplasty with and Without Prior Knee Hardware

**DOI:** 10.1007/s43465-025-01575-3

**Published:** 2025-10-03

**Authors:** Khaled A. Elmenawi, Nickelas Huffman, Cleveland Clinic Adult Reconstruction Research Group, Shujaa T. Khan, Ignacio Pasqualini, Lakshmi S. Gudapati, Chao Zhang, Matthew E. Deren, Peter A. Surace, John P. McLaughlin, Trevor G. Murray, Viktor E. Krebs, Robert M. Molloy, Nicolas S. Piuzzi

**Affiliations:** 1 Department of Orthopedic Surgery, Orthopedic and Rheumatology Institute Cleveland Clinic, 9500 Euclid Ave, Cleveland, OH A4144195 USA; 2 Department of Biomedical Engineering, Cleveland Clinic, Cleveland, OH USA

**Keywords:** Total knee arthroplasty, Prior hardware, Conversion total knee arthroplasty, Open reduction and internal fixation, PROMs

## Abstract

**Purpose:**

Total knee arthroplasty (TKA) in patients with prior knee hardware presents unique surgical challenges and may increase complication risks. However, data on patient-reported outcome measures (PROMs) and healthcare utilization in this population are limited. This study aimed to compare clinically significant improvements in 1-year PROMs, healthcare utilization, and survivorship free from reoperation at 1 year among three groups: plate and screws, intramedullary nail, and control.

**Methods:**

From 2016 to 2023, 51 TKAs with prior knee hardware (36 plate and screws, 15 intramedullary nails) were matched to 102 controls. PROMs included 1-year Knee injury Osteoarthritis Outcome Score (KOOS) for Pain, Joint Replacement (JR), and Physical function Shortform (PS), assessed by achievement of Minimal Clinically Important Difference (MCID) and Patient Acceptable Symptom State (PASS). Healthcare utilization metrics included length of stay (LOS), 90-day readmissions, emergency department (ED) visits, discharge disposition (DD), and utilization of stems. Survivorship was determined by 1-year mortality and reoperations.

**Results:**

There were no significant differences in 1-year KOOS-Pain (*p* = 0.94), PS (*p* = 0.91), and JR (*p* = 0.9). Rates of achieving MCID and PASS thresholds were similar. Differences in LOS (*p* = 0.15), 90-day readmissions (*p* = 0.61), ED visits (*p* = 0.11), DD (*p* = 0.13), and stem use (*p* > 0.05) were insignificant. Mortality (*p* = 1) and reoperation rates (*p* = 0.69) were also comparable.

**Conclusions:**

TKA in patients with prior knee hardware yielded similar 1-year PROMs, healthcare utilization, and survivorship to those without hardware. Understanding management strategies is crucial to optimize outcomes in this complex patient population.

## Introduction

Total knee arthroplasty (TKA) is the standard treatment to alleviate pain and improve joint function in patients with end-stage knee osteoarthritis [1]. TKA is sometimes performed in patients who have previously underwent surgical instrumentation, such as ligament reconstruction, deformity correction, or fracture fixation [2, 3]. These procedures often leave behind implanted hardware with scar tissue, altered anatomy, and bone defects that present unique technical challenges for surgeons during TKA [4–8], predisposing patients to worse postoperative outcomes and accumulating more costs [9–12].

Despite the additional complexities, risks, and increased cost when performing TKA in patients with prior knee hardware, there is a paucity of data examining patient-reported outcomes measures (PROMs), and associated healthcare utilization, particularly in patients who underwent open reduction and internal fixation (ORIF) before TKA. Understanding the impact of prior ORIF on the recovery journey, utilization of healthcare resources, and PROMs after TKA provides the foundation for establishing high-value patient-centered care.

Therefore, the current study aims to compare patients with prior knee hardware following ORIF undergoing TKA to a control group without prior knee hardware in terms of 1) clinically significant improvement in 1-year PROMs [Minimal Clinically Important Difference (MCID) and Patient Acceptable Symptom State (PASS)], 2) healthcare utilization parameters [length of stay (LOS), discharge disposition (DD), 90-day readmission, 90-day emergency department (ED) visits, and utilization of stemmed implants], and 3) 1-year survivorship free from reoperation and mortality.

## Methods

### Study Design and Data Collection

**Fig. 1:**
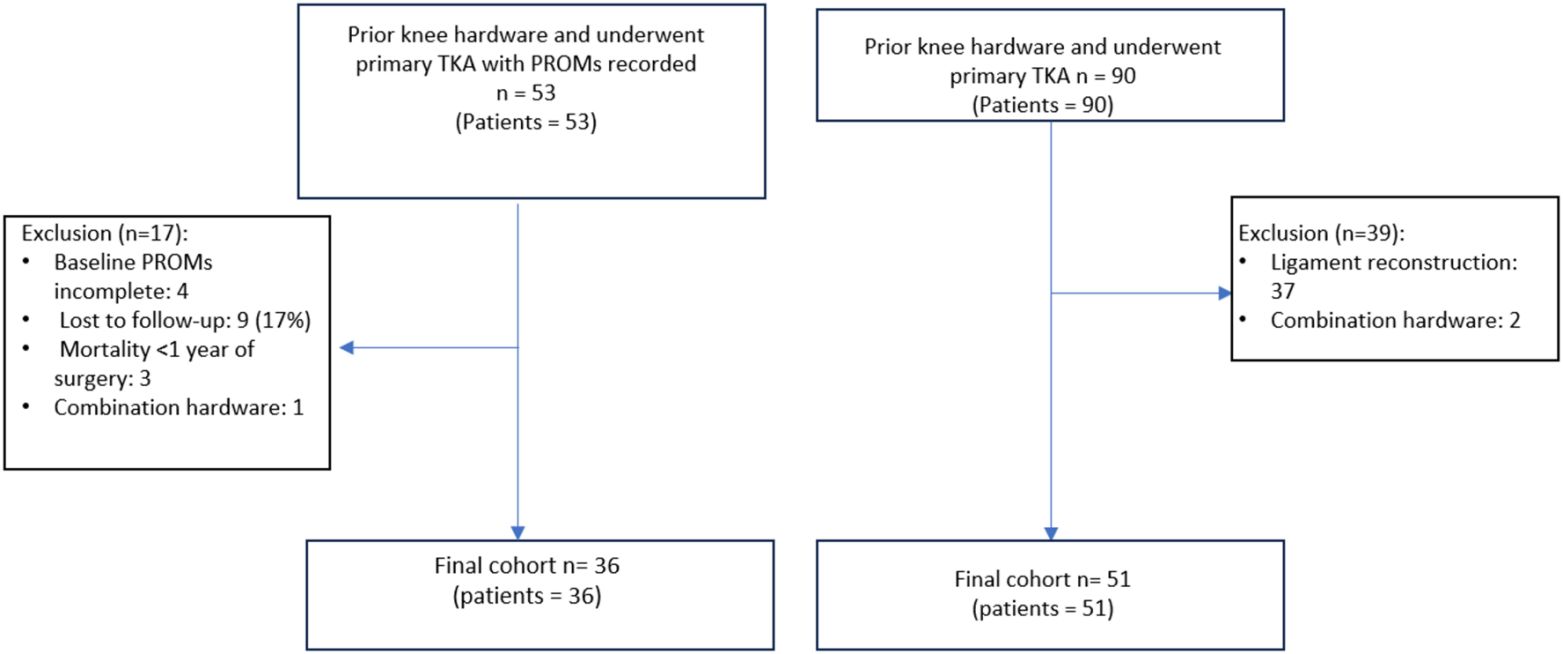
Strengthening the Reporting of Observational studies in Epidemiology (STROBE) inclusion/exclusion diagram

A total of 90 primary TKA with prior hardware between January 2016 and March 2023 from a single healthcare system were prospectively enrolled for potential inclusion. Out of 90 TKA procedures, 51 patients with prior knee hardware from ORIF (36 plates and screws, 15 intramedullary nails) and complete healthcare utilization data were matched by age and sex to 102 controls who had TKA without prior hardware (Fig. 1). These two groups were compared for healthcare utilization metrics only. Additionally, 36 patients with prior knee hardware from ORIF (24 with plates and screws, 12 with intramedullary nails) and complete baseline and 1-year PROMs were matched with 63 controls who had TKA without prior hardware (Fig. 1). These two groups were compared for PROMs only. Institutional Review Board (IRB) approval was obtained prior to beginning the study. The project was reviewed and approved by our IRB to ensure compliance with ethical standards. The review was conducted in accordance with the principles outlined in the Declaration of Helsinki and adhered to the Health Insurance Portability and Accountability Act (HIPAA) regulations for the protection of patient privacy and confidentiality.

Data were collected in our Orthopaedic Minimal Data Set Episode of Care (OME) system that captures patient-reported outcome measures (PROMs), surgical details, and demographics for consecutive patients via Research Electronic Data Capture (REDCap) software for almost all patients [13–16]. Data recorded included age, sex, body mass index (BMI), education, area deprivation index (ADI), race, smoking status, Charlson Comorbidity Index (CCI), insurance type, 1-year PROMs, indication for surgery, TKA implant characteristics, baseline opioid overdose risk (NARX) score, and healthcare utilization parameters, such as LOS, 90-day readmissions, 90-day ED visits, DD, and utilization of stemmed implants. PROMs included: 1-year Knee injury and Osteoarthritis Outcome Score (KOOS) for Pain, Physical function Shortform (PS), and Joint Replacement (JR). Achievement of the MCID and PASS were assessed for each measure. MCID thresholds were 8 for KOOS-Pain and KOOS-PS and 6.8 for KOOS-JR. PASS thresholds were 77.7 for KOOS-Pain and 70.3 for both KOOS-PS and KOOS-JR [17, 18]. Survivorship was assessed using 1-year reoperations, and 1-year mortality.

The ADI measures socioeconomic disadvantage across various regions by analyzing factors such as income, education, employment, and housing conditions. Elevated ADI scores suggest higher levels of deprivation, meaning that individuals in these areas face more substantial economic and social challenges [19–21]. The NARX score assesses the risk of prescription drug overdose, with a scale from 0 (indicating no drug use) to 999 (indicating the highest risk). This score is determined through an algorithm that examines data on both current and past prescription use, particularly focusing on opioids, sedatives, and stimulants [22–24].

### Data Analysis

Continuous variables were displayed using median and (IQR), while categorical variables were displayed using counts and percentages. Normality assumptions were assessed using the Shapiro–Wilk test. The control cohort was selected based on exact matching using Gender and nearest neighbor matching using Age. To explore differences between the groups for continuous variables, the Wilcoxon–Mann–Whitney test was applied, while the Chi-squared test or Fisher exact test was employed for categorical variables. All tests were two-sided, assuming a significant level of 0.05.

## Results

### Baseline Demographics

A total of 153 TKA cases (51 prior knee hardware, 102 no hardware controls) from January 2016 to March 2023 were included. Median (IQR) for age was 57 (50;67), 56.5 (49;68), and 58 (52;65), median BMI (IQR) was 32 (28;36), 29 (26;33), and 28 (26;36) for controls, plate and screws, and intramedullary nail cases, respectively. Overall, 63% (*n* = 96) of our cohort were Women. The indication for surgery was osteoarthritis in 91% (*n* = 91), 60% (*n* = 9), and 50% (*n* = 18) of patients without prior hardware, patients with intramedullary nail, and patients with plate and screws, respectively (Table 1).

**Table 1:** Summary table for characteristics and clinical variables for patients with and without prior knee hardware

Variable	Level	No hardware (*n* = 102)	Plate and screws (*n* = 36)	Intramedullary nail (*n* = 15)	*P* value	Participant number
Age		57.0 [49.2;67.0]	56.5 [49.0;68.0]	58.0 [52.0;64.5]	0.934	153
Sex	Women	64 (62.7%)	23 (63.9%)	9 (60.0%)	0.966	153
BMI		31.6 [28.0;35.5]	29.0 [25.8;32.7]	27.9 [25.8;35.5]	0.141	138
Education		14.0 [12.0;16.0]	14.0 [12.0;16.0]	13.0 [12.0;15.0]	0.872	138
ADI		60.0 [34.8;83.0]	64.5 [52.5;83.0]	66.0 [55.5;88.5]	0.245	143
Race	White	78 (78.8%)	29 (85.3%)	11 (84.6%)	0.682	146
	Non-white	21 (21.2%)	5 (14.7%)	2 (15.4%)		
Smoking	Never	50 (54.9%)	17 (53.1%)	11 (73.3%)	0.602	138
	Quit	24 (26.4%)	7 (21.9%)	2 (13.3%)		
	Current	17 (18.7%)	8 (25.0%)	2 (13.3%)		
CCI		0.00 [0.00;1.00]	0.00 [0.00;1.00]	1.00 [0.50;2.00]	**0.042**	150
Insurance	Other	49 (52.7%)	21 (65.6%)	5 (38.5%)	0.216	138
	Medicaid/Medicare	44 (47.3%)	11 (34.4%)	8 (61.5%)		
Anesthesia	General	22 (21.8%)	22 (61.1%)	5 (33.3%)	**0.001**	152
	Spinal	78 (77.2%)	13 (36.1%)	8 (53.3%)		
Diagnosis	Non-OA	9 (9.00%)	18 (50.0%)	6 (40.0%)	** < 0.001**	151
	OA	91 (91.0%)	18 (50.0%)	9 (60.0%)		
Baseline NARX (risk)		110 [0.00;190]	270 [190;310]	260 [220;350]	** < 0.001**	89

Bold values indicate statistical significane

Continuous variables presented as median and interquartile ranges, while categorical variables presented as numbers and percentages

*BMI* body mass index, *ADI* area deprivation index, *CCI* Charlson comorbidity index, *NARX* opioid overdose risk score

Outcomes of interest were to compare healthcare utilization parameters, 1-year PROMs, achievement of PASS and MCID thresholds, 1-year reoperations, and 1-year mortality between three groups: plate and screws, intramedullary nail, and controls without hardware (Table 1).

### 1-Year PROMs

Overall, there were no statistically significant differences in the 1-year KOOS for Pain (*p* = 0.94), PS (*p* = 0.91), and JR (*p* = 0.90) scores across the three groups (Table 2). Similarly, achievement of MCID-Pain (*p* = 0.34), MCID-PS (*p* = 0.52), MCID-JR (*p* = 0.58), PASS threshold (*p* = 0.61), PASS-Pain (*p* = 0.76), PASS-PS (*p* = 0.46), and PASS-JR (*p* = 1) were the same between the three groups (Table 2).

**Table 2:** Summary table for 1-year outcomes for patients with and without prior knee hardware

Variable	Level	No hardware (*n* = 63)	Plate and screws (*n* = 24)	Intramedullary nail (*n* = 12)	*P* value	Participant number
1-Year KOOS-Pain		86.1 [76.4;94.4]	86.1 [69.4;95.1]	88.9 [68.7;97.2]	0.947	99
1-Year KOOS-PS		75.1 [69.2;85.2]	73.8 [66.4;86.3]	78.0 [70.3;85.4]	0.917	98
1-Year KOOS-JR		73.3 [68.3;84.6]	76.3 [67.1;88.3]	76.3 [69.7;84.6]	0.909	90
MCID-Pain	Improved	59 (93.7%)	20 (83.3%)	11 (91.7%)	0.345	99
	Failure	4 (6.35%)	4 (16.7%)	1 (8.33%)		
MCID-PS	Improved	56 (88.9%)	19 (79.2%)	10 (90.9%)	0.527	98
	Failure	7 (11.1%)	5 (20.8%)	1 (9.09%)		
MCID-JR	Improved	53 (94.6%)	19 (86.4%)	10 (90.9%)	0.585	89
	Failure	3 (5.36%)	3 (13.6%)	1 (9.09%)		
PASS-Pain	Improved	47 (74.6%)	16 (66.7%)	8 (66.7%)	0.762	99
	Failure	16 (25.4%)	8 (33.3%)	4 (33.3%)		
PASS-PS	Improved	47 (74.6%)	15 (62.5%)	9 (81.8%)	0.460	98
	Failure	16 (25.4%)	9 (37.5%)	2 (18.2%)		
PASS-JR	Improved	39 (69.6%)	16 (69.6%)	8 (72.7%)	1.000	90
	Failure	17 (30.4%)	7 (30.4%)	3 (27.3%)		
PASS Threshold		53 (84.1%)	22 (91.7%)	11 (91.7%)	0.610	99

Continuous variables presented as median and interquartile ranges, while categorical variables presented as numbers and percentages

*KOOS* knee injury osteoarthritis outcome score, *PS* physical function shortform, *JR* joint replacement, *MCID* minimal clinically important difference, *PASS* patient acceptable symptom state

### Healthcare Utilization and Survivorship

Overall, no statistically significant differences were observed between the three groups in LOS (*p* = 0.15), 90-day readmissions (*p* = 0.61), 90-day ED visits (0.11), and DD (*p* = 0.13). Survivorship free from reoperations at 1-year (*p* = 0.69), and 1-year mortality (*p* = 1) were also similar between the three groups. Polyethylene type (*p* = 0.6), polyethylene thickness (*p* = 0.88), and stem utilization (*p* = 0.05) did not differ among the three groups (Table 3). There was a significant difference in operative time among the three groups (*p* < 0.01). The plate screws group had the longest operative time (median = 3 h, IQR 3–4), followed by the intramedullary nail group (median = 3 h, IQR 2–3), and the no hardware group (median = 2 h, IQR 2–3). A statistically significant difference was found between the three groups in hardware management (*p* < 0.01). Out of 36 patients in the plate and screws group, 23 patients (64%) underwent hardware removal before TKA, 6 patients (16.7%) underwent hardware removal at the time of TKA, and 7 patients (19%) underwent partial hardware removal. In the intramedullary nail group (*n* = 15), 8 patients (53%) underwent hardware removal before TKA, 5 patients (33%) underwent partial hardware removal, and 2 patients (13%) underwent hardware removal at the time of TKA (Table 3). Overall, 1-year reoperation rate was the same for the three groups (*p* = 0.69).

**Table 3:** Summary table for healthcare and implant utilization for patients with and without prior knee hardware

Variable	Level	No hardware (*n* = 102)	Plate and screws (*n* = 36)	Intramedullary nail (*n* = 15)	*P* value	Participant number
Operative time		2.33 [2.05;2.65]	3.00 [2.61;3.90]	2.72 [2.42;3.07]	** < 0.001**	152
Hardware removal	Remove prior to surgery	0	23 (63.9%)	8 (53.3%)	**0.002**	51
	Remove at surgery	0	6 (16.7%)	2 (13.3%)		
	Partial retention	0	7 (19.4%)	0		
	Total retention	0	0	5 (33.3%)		
LOS		1.00 [1.00;2.00]	1.00 [1.00;3.00]	1.00 [1.00;3.00]	0.159	152
DD	Home/home health care	95 (94.1%)	34 (94.4%)	12 (80.0%)	0.134	152
	Non-home	6 (5.94%)	2 (5.56%)	3 (20.0%)		
90-Day readmission		4 (3.96%)	3 (8.33%)	1 (6.67%)	0.617	152
90-Day ED visits		10 (9.80%)	8 (22.2%)	1 (6.67%)	0.117	153
1-Year reoperation		10 (9.80%)	5 (13.9%)	1 (6.67%)	0.698	153
1-Year mortality		1 (0.98%)	0 (0.00%)	0 (0.00%)	1.000	153
Polyethylene type	CR	26 (44.8%)	2 (28.6%)	3 (50.0%)	0.609	71
	CS	29 (50.0%)	4 (57.1%)	2 (33.3%)		
	MC	3 (5.17%)	1 (14.3%)	1 (16.7%)		
Polyethylene thickness	14 +	5 (4.90%)	3 (8.57%)	1 (6.67%)	0.882	152
	9–13	97 (95.1%)	32 (91.4%)	14 (93.3%)		
Stem	Both	5 (27.8%)	14 (63.6%)	5 (83.3%)	0.051	46
	Femoral	1 (5.56%)	2 (9.09%)	0 (0.00%)		
	Tibial	12 (66.7%)	6 (27.3%)	1 (16.7%)		

Bold values indicate statistical significane

Continuous variables presented as median and interquartile ranges, while categorical variables presented as numbers and percentages

*LOS* length of stay, *DD* discharge disposition, *ED* emergency department

## Discussion

Performing TKA in the presence of prior knee hardware is often challenging due to altered anatomy, bone loss, and scar tissue [4–6]. In turn, these patients may have an increased risk of postoperative complications, which may predispose them to worse PROMs and increase the utilization of healthcare resources [9, 10]. Our study compared three groups (plate and screws, intramedullary nail, no hardware controls) in terms of 1-year PROMs, healthcare utilization, and survivorship free from reoperation and mortality at 1 year. Patients with prior knee hardware who underwent TKA had similar 1-year PROMs and achievement of PASS, and MCID thresholds to those without prior knee hardware. Furthermore, LOS, 90-day readmissions, 90-day ED visits, and DD were similar between the three groups. Similarly, 1-year reoperations, 1-year mortality, polyethylene type, polyethylene thickness, and stem utilization were the same for the three groups. However, patients with prior knee hardware had a statistically significant longer operative time.

Our finding of no difference in 1-year PROMs between patients undergoing TKA with and without prior hardware aligns with recent reports. Scott et al. compared Physical Component Summary (PCS) and Mental Component Summary (MCS) of the Short Form 12-Item Health Survey (SF-12), and Oxford Knee Score (OKS) in 93 controls matched for age and sex with 31 tibial plateau fractures, and found that postoperative MCS and PCS scores at 1 year, and OKS score at 6 months, 1 year, and 5 years, were similar between the two groups [25]. Manzotti et al., found similar Knee Society Score (KSS) and Western Ontario and McMaster Universities Osteoarthritis Index (WOMAC) when comparing post-traumatic patients with retained hardware (*n* = 16) to patients without any retained hardware (*n* = 16) undergoing TKA [26]. Lizaur-Utrilla et al. evaluated PROMs between matched controls (*n* = 58) and a group of tibial plateau fractures (*n* = 29) and found similar KSS, WOMAC, and SF-12 physical and mental scores [27]. However, these studies did not report KOOS scores, or achievement of MCID and PASS thresholds, which limits the applicability of their results in clinical practice. Our findings, in conjunction with previous reports on PROMs, strongly support that patients with prior knee hardware experience comparable improvements in pain relief, function, and quality of life to those without prior knee hardware after TKA. It is worth noting that a recent study comparing KOOS-JR between patients with (*n* = 52) and without (*n* = 208) prior knee hardware following tibial plateau fractures revealed lower mean scores in those with prior knee hardware (47 vs 66, *p* = 0.01) [28]. However, their reported lower KOOS-JR scores could be attributed to a high percentage of patients with retained hardware (54%) and the inclusion of only tibial plateau fracture patients [28].

The impact of pre-existing knee hardware on healthcare utilization following TKA is not yet well defined. However, most studies observed a higher risk of postoperative complications, which subsequently leads to increased healthcare resource utilization in these patients. For example, Bergen et el. found a higher rate of 90-day readmissions, 90-day ED in TKA with prior knee hardware (*n* = 109) relative to those without (*n* = 109), but similar LOS and DD between the two groups [4], and Kreitz et al. revealed similar LOS and DD, but twice the rate of reoperations and 90-day readmissions in patients with prior knee hardware (*n* = 63) in comparison to those without (*n* = 189) when undergoing TKA [29]. This highlights the necessity of introducing a distinct Current Procedural Terminology (CPT) code specifically for knee conversion arthroplasty, to adequately compensate surgeons for the increased time and complexity involved in such procedures [29]. However, in an investigation of 30 patients, Malhotra et al. found similar 30-day reoperation rates, and LOS between patients with and without knee hardware who underwent TKA. The present study observed no difference in healthcare utilization between patients with and without prior knee hardware who underwent TKA. These inconsistencies are predominantly attributed to differences in surgical techniques, variations in implant types, and patient-specific factors that may affect the postoperative outcomes of individuals undergoing TKA.

Prior reports have assessed staged versus concurrent hardware removal at time of TKA. For example, Bergen et al. evaluated 90-day outcomes between patients who had hardware removal before TKA (*n* = 51) and those who had hardware removal during TKA (*n* = 58), and found no statistically significant differences in mechanical complications (9 vs 4%, *p* = 0.4) infections (9 vs 7%, *p* = 0.7), 90-day readmissions (16 vs 13%, *p* = 0.7), or DD (*p* = 0.2) [4]. However, their study included a heterogeneous group of different hardware types. In a more detailed investigation, Smith et al. conducted a review of 155 TKAs, comparing staged versus concurrent hardware removal with respect to complication, reoperation, and revision rates over multiple time intervals (90 days, 1 year, 2 years, and 4 years) according to the type of hardware involved (including plates, nails, rods, screws, buttons, and wires). The study found no statistically significant differences between the two approaches [30]. In the present study, a comparison between staged and concurrent hardware removal was not applicable due to the small cohort size.

The findings of this study underscore the importance of refining our understanding of TKA outcomes in patients with prior knee hardware, as this population represents a growing subset of candidates for joint replacement. Beyond the clinical implications, there are meaningful considerations for healthcare policy and cost [12, 31]. Procedures involving prior hardware are technically more complex, often requiring increased operative time and potentially higher resource utilization, yet current reimbursement frameworks may not adequately reflect this added complexity [31]. Future studies should therefore extend follow-up to evaluate long-term survivorship, revision risk, and functional recovery in these patients while also incorporating detailed cost analyses. Large, multicenter investigations and registry-based studies could provide the statistical power necessary to capture rare complications and better inform value-based care strategies. Moreover, health policy research is warranted to evaluate the potential benefit of establishing a distinct reimbursement codes for conversion TKA, which could align reimbursement more closely with procedural difficulty and resource demand. By integrating outcomes research with economic and policy perspectives, future work can help ensure that care delivery remains both equitable and sustainable in this challenging clinical context.

This study presents several potential limitations that should be acknowledged. First, the relatively small sample size may limit the generalizability of the findings and reduce the statistical power needed to detect more subtle differences. Second, inherent biases may have arisen during the data collection process. Third, the study did not capture long-term survivorship data beyond 1 year, restricting the ability to assess the durability of outcomes and any late-emerging complications or revisions over an extended follow-up period. Fourth, we did not separately compare differences in outcomes between patients who had plate and screws and those who had no hardware. This comparison is more clinically relevant and future studies should aim to compare these groups. Despite these limitations, this study presents a valuable assessment of clinically meaningful improvements in 1-year PROMs, along with healthcare utilization and 1-year survivorship, for individuals undergoing TKA with and without prior knee hardware from ORIF.

## Conclusions

Patients undergoing primary TKA in the setting of prior knee hardware achieve comparable 1-year PROMs, survivorship, and healthcare utilization outcomes to those without hardware, despite the increased technical complexity of these procedures. These findings reinforce that favorable short-term outcomes are achievable in this challenging population when appropriate surgical planning and execution are undertaken. Nonetheless, the presence of prior hardware introduces unique considerations, including altered anatomy, potential bone loss, and extended operative times, which underscore the need for tailored perioperative and intraoperative management strategies. Optimizing these approaches is essential not only to minimize complications but also to sustain functional recovery and long-term implant durability. Future investigations should prioritize larger, multicenter studies with extended follow-up to evaluate revision risk, implant survivorship, and cost-effectiveness. Additionally, integrating outcomes research with healthcare policy considerations—such as the potential development of a dedicated CPT code for conversion TKA—may better align reimbursement with the procedural demands of these cases. Collectively, these efforts will be critical in advancing evidence-based care and ensuring high-value, patient-centered outcomes for this growing cohort of TKA candidates.

## Data Availability

The datasets generated and/or analyzed during the current study are not publicly available due to privacy concerns/ethical restrictions, but are available from the corresponding author on reasonable request.
